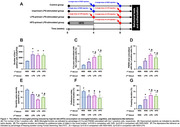# Microglial priming induced by high‐fat diet consumption causes complement C1q‐mediated synaptic elimination leading to cognitive decline and depressive‐like behavior

**DOI:** 10.1002/alz.085030

**Published:** 2025-01-03

**Authors:** Titikorn Chunchai, Thirathada Chinchapo, Hiranya Pintana, Patcharapong Pantiya, Busarin Arunsak, Suriphan Donchada, Nattayaporn Apaijai, Wasana Pratchayasakul, Nipon Chattipakorn, Siriporn C Chattipakorn

**Affiliations:** ^1^ Neurophysiology Unit, Cardiac Electrophysiology Research and Training Center, Faculty of Medicine, Chiang Mai University, Chiang Mai Thailand; ^2^ Center of Excellence in Cardiac Electrophysiology Research, Chiang Mai University, Chiang Mai Thailand; ^3^ Department of Physiology, Faculty of Medicine, Chiang Mai University, Chiang Mai Thailand; ^4^ Office of Research Administration, Chiang Mai University, Chiang Mai Thailand; ^5^ Neurophysiology unit, Cardiac Electrophysiology Research and Training Center, Faculty of Medicine, Chiang Mai University, Chiang Mai Thailand; ^6^ Cardiac Electrophysiology Research and Training Center, Faculty of Medicine, Chiang Mai University, Chiang Mai Thailand; ^7^ Department of Oral Biology and Diagnostic Sciences, Faculty of Dentistry, Chiang Mai University, Chiang Mai Thailand

## Abstract

**Background:**

Microglia play an important role in immune memory. Lipopolysaccharide (LPS) triggers immune memory and primes microglia, resulting in brain pathologies and brain dysfunction following a second stimulus (1, 2). An increase in the C1q/ PSD95 expressions within microglia and excessively synaptic pruning were observed in mouse model of Alzheimer’s disease (3). However, the effects of microglial priming induced by high‐fat diet (HFD) on microglial function, synaptic plasticity, cognition, and depressive‐like behavior after the secondary LPS stimulation have not been investigated.

**Method:**

Twenty‐four male Wistar rats were randomly assigned to be three primed conditions to receive either normal saline (NSS: intraperitoneal injection (i.p., as single dose)), LPS (0.5 mg/kg, i.p. as a single dose), or 4 weeks of 60% HFD consumption. Then at week 8, rats in each condition were divided into 2 groups. Each group was received the single dose of second stimulus of either NSS (i.p.) or LPS (0.5 mg/kg, i.p.). After 12‐16 hrs from second stimulus, the cognitive function and depressive‐like behavior were determined in all rats. Then animals were euthanized, and brains were removed for further analysis.

**Result:**

All rats receiving LPS injection as the second stimulation with or without primed condition, equally increased microglial numbers, when compared to rats without any LPS stimulation or control rats (p<0.05, Figure 1). Interestingly, rats receiving second LPS injection increased C1q/PSD95 colocalized with microglia, decreased dendritic spine density, and increased freezing duration, when compared to the control rats (p<0.05, Figure 1). LPS‐stimulated rats with both LPS and HFD‐primed conditions showed the highest levels of these parameters (p<0.05, Figure 1). In addition, All LPS‐stimulated rats with or without primed conditions exhibited cognitive decline, when compared to the control rats (p<0.05, Figure 1).

**Conclusion:**

Our findings suggest that only 4‐week HFD consumption effectively primed microglia to a similar extent as primed with a single dose of LPS injection, leading to excessive synaptic engulfment, as well as cognitive decline and depressive‐like behavior in the second LPS stimulation after 4‐week of HFD withdrawal period.